# Exploring carbon catenoids and their applications for encapsulation of carbon nanostructures

**DOI:** 10.1371/journal.pone.0310740

**Published:** 2024-09-26

**Authors:** Panyada Sripaturad, Ngamta Thamwattana, Kyle Stevens, Duangkamon Baowan

**Affiliations:** 1 Department of Mathematics, Faculty of Science, Mahidol University, Bangkok, Thailand; 2 School of Information and Physical Sciences, University of Newcastle, Callaghan, NSW, Australia; 3 Centre of Excellence in Mathematics, CHE, Bangkok, Thailand; Saveetha University - Poonamallee Campus: SIMATS Deemed University, INDIA

## Abstract

Carbon nanostructures of various shapes are among materials that have been extensively studied due to their unique chemical and physical properties. In this paper, we propose a new geometry of carbon nanostructures known as molecular carbon catenoid to compare with theoretical catenoid found from minimising the Willmore energy functional. Since applications of this structure include electron and molecular transport, this paper mathematically models the energetic behaviour of an atom and a spherical molecule entering a catenoid using the Lennard-Jones potential and a continuum approach. The suction energy is also obtained to determine the size of catenoid suitable for encapsulation of various structures. Results shown for theoretical catenoid using continuum modelling approach are found to be in good agreement with numerical simulations for molecular carbon catenoid.

## 1 Introduction

**Fig 1:**
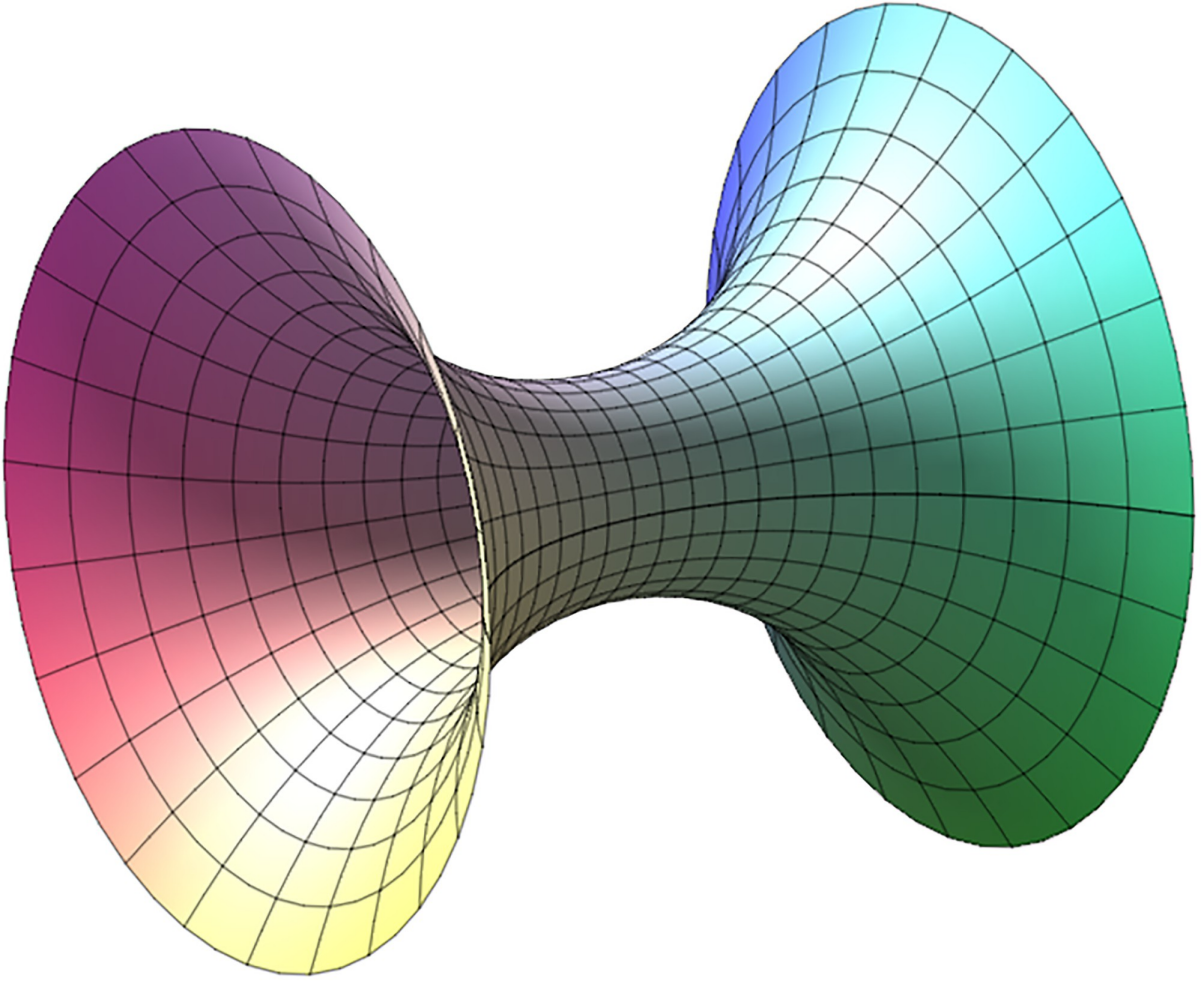
A catenoid surface—An absolute minimiser of the Willmore energy functional.

Driven by their unique electronic, chemical and mechanical properties, carbon nanostructures have been extensively studied for potential uses in various applications, including medical devices, water treatment and desalination [1–4]. Development of new shapes and geometries of carbon materials to further improve their properties also receives much attention [5–7]. One structure that is of particular interest is a catenoid surface as shown in Fig 1. In Sripaturad et al. [8], catenoid is shown to be an absolute minimiser of the Willmore energy. Therefore, catenoids are used as a minimal energy surface to join between nanostructures to form novel configurations, leading to new structures with enhanced functionalities [8–10].

The encapsulation and penetration of nanoparticles or ions through nanoscale channels have been extensively studied using various methods including mathematical modelling [7, 11, 12] and molecular dynamics simulations [13, 14]. Similarly, the oscillatory behaviour of carbon nanostructures has been widely investigated including carbon atoms and fullerenes inside carbon nanotubes [15], double-walled carbon nanotubes [16], carbon atoms inside nanotori [17], and carbon nanotori inside carbon nanotubes [18]. These findings motivate our study here to explore a novel carbon nanostructure (i.e. carbon catenoid) and analyse its encapsulation energy for uses as molecular transport.

As catenoids may be used as an artificial channels connecting two structures for gas storage or ion channels [9, 10, 19, 20], it is important to understand the mechanics of atomic and molecular interactions with catenoids. This may also be useful for the design of catenoids to be used as molecular selective filters [21, 22]. Mathematical models have been employed to analyse the problems of nanostructure encapsulation, determining both the geometric feasibility and structural stability of the systems. Cox et al. [23] investigate the mechanical behaviour of single-walled carbon nanotubes interacting with both atoms and C_60_ fullerenes. Sarapat et al. [24] use a similar approach to Cox et al. [23] to describe the energy landscape of selective filters modeled as nanocones. Furthermore, Baowan et al. [22] study the ion transport through cylindrical and hourglass-shaped PET channels. These studies utilise the Lennard-Jones function to calculate the potential energies between interacting structures and employ the concept of suction energy to determine the critical dimension for which atoms or molecules can be encapsulated into a nanostructure.

In this paper, we construct for the first time a molecular carbon catenoid shown in Section 3 to compare with theoretical catenoid originated from minimising the Willmore energy. We then investigate the encapsulation capacity of catenoids for both atoms and fullerenes using a continuum approach and molecular dynamics simulations. Assuming catenoid and spherical fullerenes consisting solely of carbon atoms, Section 3.1 details the continuum approach for finding the interaction energy between a carbon atom and a catenoid centred at the origin. In Section 3.2, the interaction energy between a spherical fullerene and a catenoid is determined. We employ the suction energy, detailed in Section 3.3, to determine the radius at the neck of a catenoid that allows encapsulation to occur. Section 4 presents results for various catenoid sizes interacting with both atom and spherical fullerenes. Comparison of results from the continuum approach and numerical simulations performed using the large-scale atomic/molecular massively parallel simulator (LAMMPS) software package [25] are presented in Section 4.3, and finally, Section 4.3 provides summary of our findings.

## 2 Theoretical and molecular carbon catenoids

The Willmore energy is defined by

$$W=\int_{S}H^{2}dA,$$

where *H* is the mean curvature of surface *S* representing the sum of both axial and rotational curvatures and *dA* is the area element. In Sripaturad et al. [8], it was shown that the surface with *H* = 0 corresponds to catenoid surfaces, and this family of surfaces is an absolute minimiser of the Willmore energy. As a result, catenoids find their uses as minimal energy surfaces for joining between structures [8–10] and other applications in nanomagnetism [26, 27].

**Fig 2:**
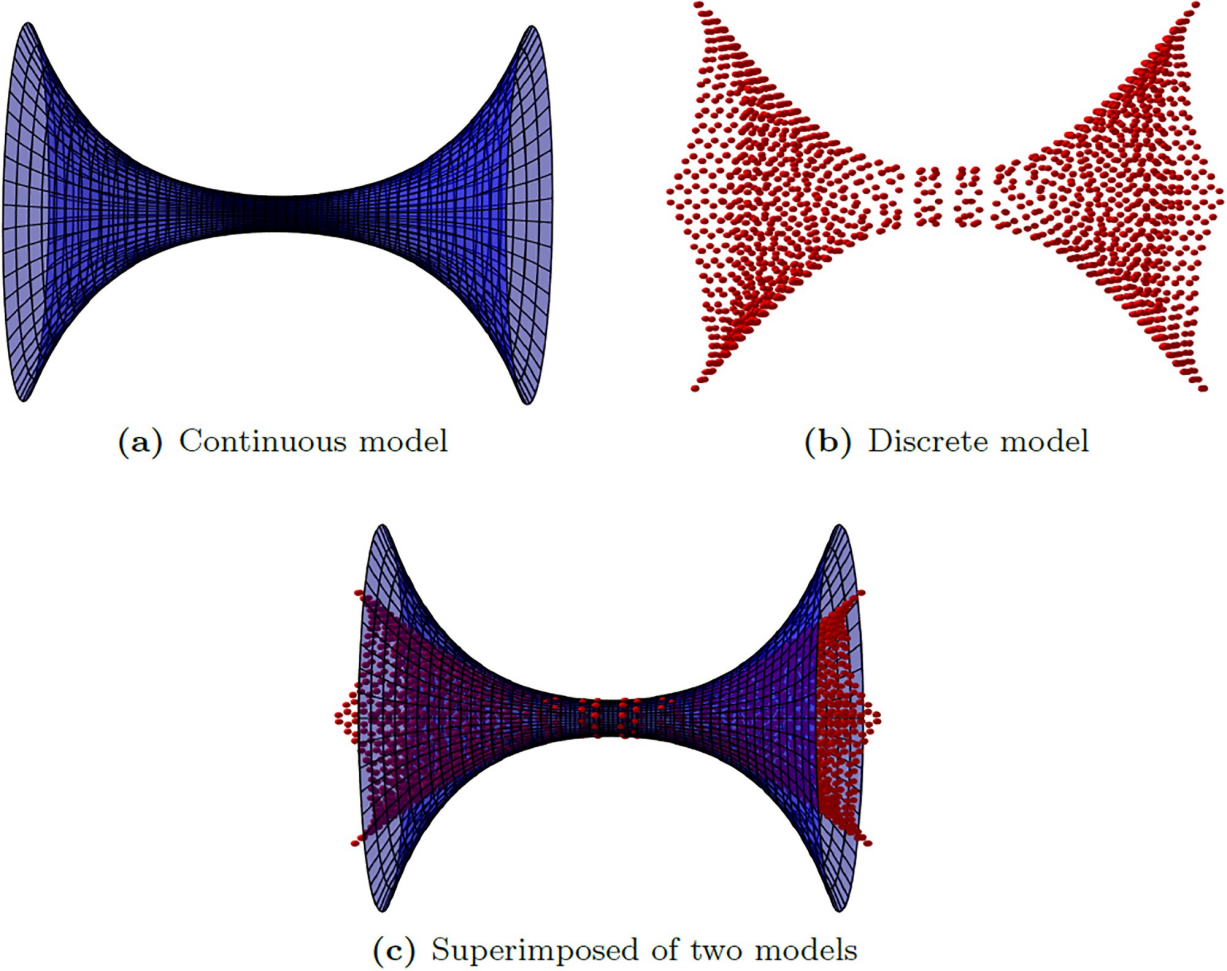
Structures of catenoids made from (a) continuous model of theoretical catenoid, (b) discrete atomistic model of molecular carbon catenoid and (c) superimposed of the two models.

The surface of the catenoid as described in [8] is given by

$$\begin{array}{c} z=\pm a\;{\text{cosh}}^{-1}(\frac{r}{a}), \end{array}$$ (1)

where *a* is the radius at the neck of the catenoid at *z* = 0. The plus (minus) sign indicates the upper (lower) half of the catenoid. Due to its symmetry, we have *r* = *a* cosh(*z*/*a*) for both upper and lower parts of the catenoid. We refer to a catenoid constructed from (1) as a “theoretical catenoid”, and a profile of this catenoid with *a* = 3.9144 Å is shown in Fig 2(a).

To benchmark the theoretical model of catenoid, we construct a “molecular carbon catenoid” using the Avogadro 2 software package [28] (see Fig 2(b)). This structure is constructed starting from a (*n*, 0) carbon nanotube and adding layers of hexagons with regularly interspersed heptagons and pentagons. The steps involved in constructing a catenoid using Avogadro software are provided in S1 File. In this paper, we use a (10, 0) nanotube, which has the radius of 3.9144 Å, with a heptagon frequency of four every third layer. This generates a molecular structure with close conformity to the theoretical catenoid of the same radius up to roughly 8 Å either side of the centre (see Fig 2(c)). We then use LAMMPS software package [25] to perform a simulation between the constructed molecular carbon catenoid and a carbon atom. The system is simulated in a domain of size 100 Å × 100 Å × 100 Å and the Lennard-Jones pair potential is used with a cut-off distance of 14 Å. We note that this cut-off distance is far enough such that the potential energy between the two structures goes to zero after this distance. The simulation results are depicted in Section 4.

## 3 Interaction of atoms and fullerenes with theoretical catenoid

Here, we model an atom as a point, a fullerene as a spherical shell of carbon atoms and a catenoid as a homogeneous surface of carbon atoms. We use the Lennard-Jones potential function and a continuum approach to determine the interaction energy between two non-bonded structures, namely

$$\begin{array}{c} E\left(\rho \right)=\eta_{1}\eta_{2}\int_{S_{1}}\int_{S_{2}}(-\frac{A}{\rho^{6}}+\frac{B}{\rho^{12}})dS_{1}dS_{2}, \end{array}$$ (2)

where *A* and *B* are the attractive and repulsive constants, respectively, *η*_1_ and *η*_2_ are mean atomic surface densities of the two structures, and *ρ* is a typical distance between surface elements of the two interacting structures. Further, *A* = 2*ϵσ*^6^ and *B* = *ϵσ*^12^ where *ϵ* is the well-depth and *σ* is the van der Waals diameter. In this paper, we obtain the values of *ϵ* and *σ* for C-C interaction from Rappe et al. [29]. As described in [29], the non-boned parameters (or the Lennard-Jones parameters) for all carbon atoms with different hybridization, geometry or oxidation state are equivalent. We comment that the systems investigated here consist exclusively of carbon atoms with a net neutral charge, allowing for the neglect of Coulomb potential. Furthermore, as demonstrated in [30], van der Waals interactions predominate over molecular energy in the nanoscale systems, especially those of carbon nanostructures [11].

To simplify the expression in (2), we define

$$\begin{array}{c} I_{n}=\int_{S}\rho^{-2n}dS, \end{array}$$ (3)

for *n* = 3 and 6, so the total energy becomes *E*(*ρ*) = *η*_1_*η*_2_[−*AI*_3_(*ρ*) + *BI*_6_(*ρ*)]. In Section 3.1, an analytical expression for the interaction energy between an atom and a catenoid is derived. For the interaction between a fullerene and a catenoid in Section 3.2, we take a two-step approach. First, we evaluate the interaction *E*_*as*_ between a point on a catenoid and a sphere. Then, we perform a surface integral of *E*_*as*_ over the catenoid to obtain the total interaction energy *E*_*sc*_ between the sphere and the entire surface of catenoid. In Section 3.3, the suction energy is determined for both atom and fullerene.

### 3.1 Interaction energy between atom and catenoid

In this section, we first derive the interaction energy between an atom and a catenoid. Here, the atom is assumed to be located along the *z*−axis at coordinates (0, 0, *d*) and a typical point on a catenoid can be prescribed by (*x*, *y*, *z*) = (*r* cos *θ*, *r* sin *θ*, *z*) where *θ* ∈ (0, 2*π*) and from (1), *r* = *a* cosh(*z*/*a*), where *a* is the radius at *z* = 0 (the neck’s radius).

**Fig 3:**
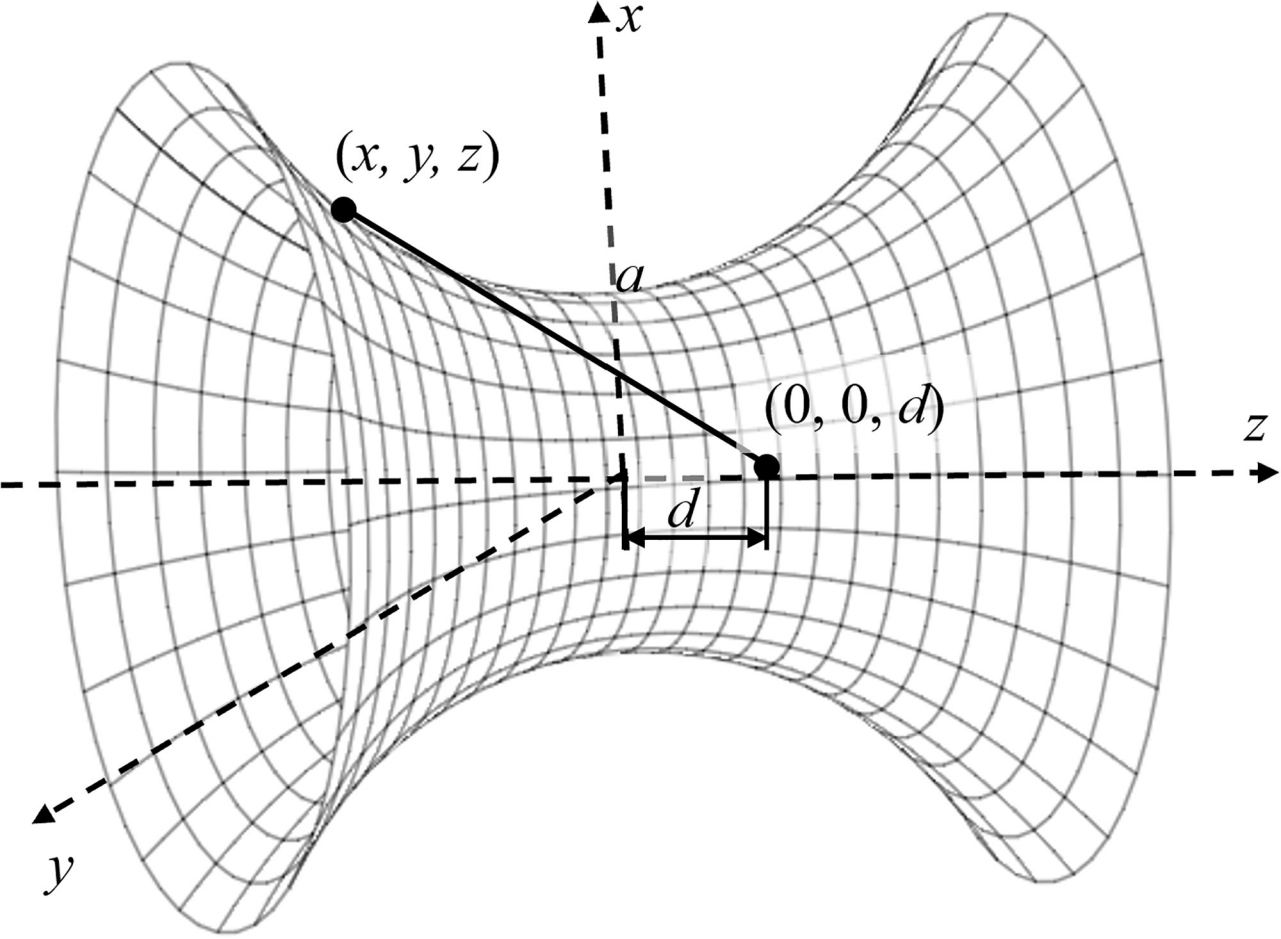
Model of atom located at coordinates (0, 0, *d*) interacting with catenoid prescribed by (*x*, *y*, *z*) = (*r* cos *θ*, *r* sin *θ*, *z*) where *r* = *a* cosh (*z*/*a*) and *a* is the radius at *z* = 0.

As shown in Fig 3, we define *ρ* to be a typical distance between the atom and a surface element of the catenoid of length 2*L*, which is given by

$$\begin{array}{c} \begin{array}{cc} \rho^{2} & ={\left[a\;\text{cosh}\left(z/a\right)\text{cos}\;\theta \right]}^{2}+{\left[a\;\text{cosh}\left(z/a\right)\text{sin}\;\theta \right]}^{2}+{\left(z-d\right)}^{2}\\ & ={\left[a\;\text{cosh}\left(z/a\right)\right]}^{2}+{\left(z-d\right)}^{2}, \end{array} \end{array}$$

where *z* ∈ (−*L*, *L*) and *θ* ∈ (0, 2*π*). Due to the symmetry of the catenoid *z* ∈ (−*L*, 0) is represented its lower part and *z* ∈ (0, *L*) is defined its upper part.

Using the formula 2 cosh^2^*x* = cosh(2*x*) + 1 and the definition of hyperbolic cosine function cosh *x* = (*e*^*x*^ + *e*^−*x*^)/2, the integral *I*_*n*_ defined by (3) becomes

$$\begin{array}{ccc} I_{n} & = & a\int_{0}^{2\pi }\int_{-L}^{L}{\left[{\left(a\;\text{cosh}\left(z/a\right)\right)}^{2}+{\left(z-d\right)}^{2}\right]}^{-n}dzd\theta \\ & = & 2\pi a\int_{-L}^{L}{\left[\frac{a^{2}}{4}\left(e^{2z/a}+e^{-2z/a}+2\right)+{\left(z-d\right)}^{2}\right]}^{-n}dz, \end{array}$$ (4)

where *L* and *a* are prescribed values. On substituting *n* = 3 and 6 into (4), the interaction energy between an atom and a catenoid in terms of the location *d* of the atom along the catenoid’s central axis is obtained and it can be written as *E*_*ac*_ = *η*_*c*_(−*AI*_3_ + *BI*_6_), where *η*_*c*_ is a mean atomic surface density of the catenoid.

### 3.2 Interaction energy between sphere and catenoid

**Fig 4:**
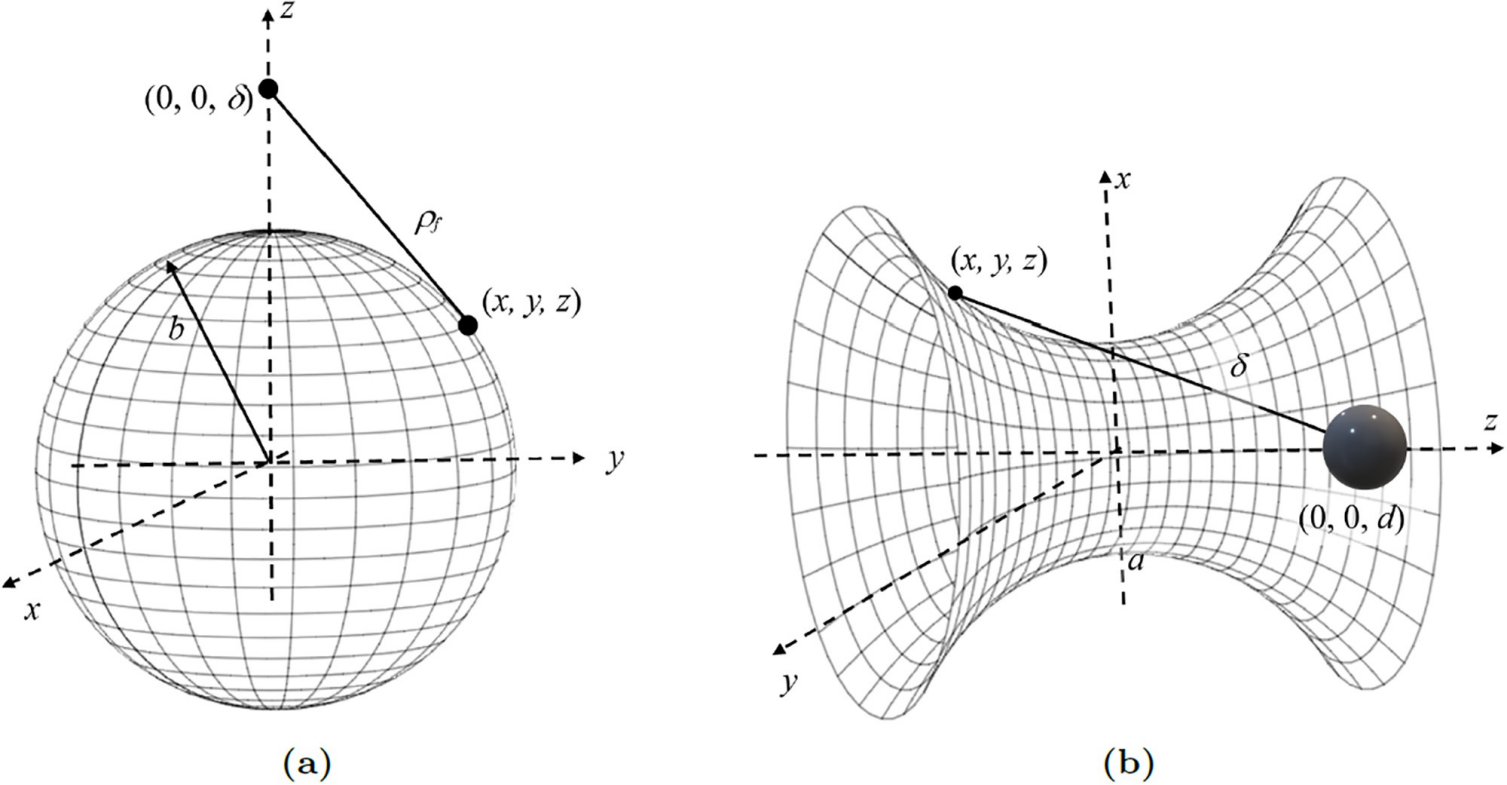
Models for (a) an atom located at (0, 0, *δ*) interacting with a sphere centred at the origin, and (b) catenoid interacting with the sphere where its centre is shifted to be located at (0, 0, *d*) and the atom from (a) is then assumed to be a typical point on the catenoid.

Here, we determine the interaction energy between a spherical shell (e.g. fullerenes) and a catenoid. To do so, we first determine the interaction energy between an atom located at (0, 0, *δ*) and a spherical shell, as shown in Fig 4(a). The spherical molecule of radius *b* is assumed to be centred at the origin with coordinates (*x*, *y*, *z*) = (*b* cos *θ* sin *φ*, *b* sin *θ* sin *φ*, *b* cos *φ*) where *θ* ∈ (−*π*, *π*) and *φ* ∈ (0, *π*). Therefore, the distance from a surface element of the sphere to the atom is given by

$$\begin{array}{ccc} \rho_{f}^{2} & = & {\left(b\;\text{cos}\;\theta \;\text{sin}\;\phi \right)}^{2}+{\left(b\;\text{sin}\;\theta \;\text{sin}\;\phi \right)}^{2}+{\left(b\;\text{cos}\;\phi -\delta \right)}^{2}\\ & = & b^{2}\;{\text{sin}}^{2}\phi +{\left(b\;\text{cos}\;\phi -\delta \right)}^{2}. \end{array}$$

Substituting *ρ*_*f*_ into (3), we have

$$\begin{array}{ccc} I_{n} & = & \int_{0}^{\pi }\int_{-\pi }^{\pi }\frac{b^{2}\;\text{sin}\;\phi }{{\left[b^{2}\;{\text{sin}}^{2}\phi +{\left(b\;\text{cos}\;\phi -\delta \right)}^{2}\right]}^{n}}\;d\theta d\phi \\ & = & 2\pi b^{2}\int_{0}^{\pi }\frac{\text{sin}\;\phi }{{\left(b^{2}-2b\delta \;\text{cos}\;\phi +\delta^{2}\right)}^{n}}\;d\phi . \end{array}$$

By making a substitution *t* = *b*^2^ − 2*bδ* cos *ϕ* + *δ*^2^, we deduce

$$\begin{array}{c} I_{n}=\frac{\pi b}{\delta \left(n-1\right)}\left(\frac{1}{{\left(\delta -b\right)}^{2\left(n-1\right)}}-\frac{1}{{\left(\delta +b\right)}^{2\left(n-1\right)}}\right). \end{array}$$ (5)

Therefore, the interaction energy between the fullerene of radius *b* and the atom becomes

$$\begin{array}{c} E_{as}=\frac{\pi b\eta_{1}}{\delta }\{-\frac{A}{2}\left[\frac{1}{{\left(\delta -b\right)}^{4}}-\frac{1}{{\left(b+\delta \right)}^{4}}\right]+\frac{B}{5}\left[\frac{1}{{\left(\delta -b\right)}^{10}}-\frac{1}{{\left(b+\delta \right)}^{10}}\right]\}, \end{array}$$

where *η*_1_ is the mean atomic surface density of the spherical shell.

Next, if we assume the atom shown in Fig 4(a) to be an arbitrary point on a surface of a catenoid, then the distance *δ* becomes the distance between the centre of the sphere and the surface of the catenoid as shown in Fig 4(b). Thus, by performing another surface integral for *I*_3_ and *I*_6_ defined by (5) over catenoid, we can obtain the interaction energy between a sphere and a catenoid. By placing fractions over common denominators, expanding and reducing to fractions in terms of powers of (*δ*^2^ − *b*^2^), the integral *I*_*n*_ given by (5) becomes

$$\begin{array}{ccc} I_{3} & = & 4\pi b^{2}\left[\frac{1}{{\left(\delta^{2}-b^{2}\right)}^{3}}+\frac{2b^{2}}{{\left(\delta^{2}-b^{2}\right)}^{4}}\right],\\ I_{6} & = & \frac{4\pi b^{2}}{5}\left[\frac{5}{{\left(\delta^{2}-b^{2}\right)}^{6}}+\frac{80b^{2}}{{\left(\delta^{2}-b^{2}\right)}^{7}}+\frac{336b^{4}}{{\left(\delta^{2}-b^{2}\right)}^{8}}+\frac{512b^{6}}{{\left(\delta^{2}-b^{2}\right)}^{9}}+\frac{256b^{8}}{{\left(\delta^{2}-b^{2}\right)}^{10}}\right]. \end{array}$$ (6)

Here, the sphere is shifted to be centred at (0, 0, *d*). The atom in Fig 4(a) is assumed to be a typical point on the surface of a catenoid as shown in Fig 4(b), and similar to Section 3.1, its coordinates are (*x*, *y*, *z*) = (*r* cos *θ*, *r* sin *θ*, *z*) where *r* = *a* cosh (*z*/*a*). Thus the distance between the centre of the sphere and the atom on the catenoid is given by

$$\begin{array}{ccc} \delta^{2} & = & {\left[a\;\text{cosh}\left(z/a\right)\text{cos}\;\theta \right]}^{2}+{\left[a\;\text{cosh}\left(z/a\right)\text{sin}\;\theta \right]}^{2}+{\left(z-d\right)}^{2}\\ & = & {\left[a\;\text{cosh}\left(z/a\right)\right]}^{2}+{\left(z-d\right)}^{2}. \end{array}$$

By defining that

$$\begin{array}{ccc} K_{m} & = & \int_{S}\frac{1}{{\left(\delta^{2}-b^{2}\right)}^{m}}dS\\ & = & a\int_{0}^{2\pi }\int_{-L}^{L}{\left[{\left(a\;\text{cosh}\left(z/a\right)\right)}^{2}+{\left(z-d\right)}^{2}-b^{2}\right]}^{-m}dzd\theta \\ & = & 2\pi a\int_{-L}^{L}{\left[\frac{a^{2}}{4}\left(e^{2z/a}+e^{-2z/a}+2\right)+{\left(z-d\right)}^{2}-b^{2}\right]}^{-m}dz, \end{array}$$

the integral *I*_*n*_ given in (6) can be written as

$$\begin{array}{ccc} I_{3} & = & 4\pi b^{2}\left(K_{3}+2b^{2}K_{4}\right),\\ I_{6} & = & \frac{4\pi b^{2}}{5}\left(5K_{6}+80b^{2}K_{7}+336b^{4}K_{8}+512b^{6}K_{9}+256b^{8}K_{10}\right). \end{array}$$ (7)

Therefore, the total interaction energy between a sphere and a catenoid is of the form *E*_*sc*_ = *η*_1_*η*_2_(−*AI*_3_ + *BI*_6_). Here, *η*_2_ is assumed be the mean surface density of the catenoid and *I*_3_ and *I*_6_ are given by (7).

### 3.3 Suction energy

Here, we consider a molecule on the negative *z*−axis entering a catenoid centred at the origin. We define the suction energy (*W*) as the total work done by the axial force *F*(*z*) = −*dE*/*dz* on a molecule entering the catenoid. Mathematically, *W* is given by

$$\begin{array}{c} W=\int_{-\infty }^{0}F\left(z\right)dz=-\int_{-\infty }^{0}\frac{dE}{dz}dz=E\left(-\infty \right)-E\left(0\right), \end{array}$$

where *E* is the total interaction energy, namely *E*_*ac*_ and *E*_*sc*_ for atom-catenoid and sphere-catenoid, respectively. The suction energy is employed to determine the critical size of a catenoid that can encapsulate an atom or a spherical molecule.

We observe that the energy at *z* = ±∞ vanishes to zero, and the suction energy may be approximated as the negative of the total interaction energy *E* at *z* = 0. This implies that if the molecule can overcome this energy barrier at *z* = 0, it can be encapsulated inside the structure. We note that *z* = 0 corresponds to *r* = *a*, which is at the neck of a catenoid. As such, using the suction energy, we can determine the radius *a* at the neck of a catenoid that maximises its interaction with an atom or a spherical molecule.

## 4 Results and discussions

Both sphere and catenoid are assumed to comprise only of carbon atoms, then its well depth is taken to be *ϵ* = 0.105 kcal/mol and its van der Waals diameter is *σ* = 3.851 Å. This is actually the value for the location of the minimum energy of the atom dimer *ρ*_*min*_, and the value of $2^{-\frac{1}{6}}\rho_{min}=3.430851$ Å is used based on the work of Rappe et al. [29]. The mean surface density of the spherical molecule is taken to be 0.3789 Å^−2^ which is that of the C_60_ fullerene [7]. For carbon catenoid, we approximate its mean atomic surface density by using the value of a flat graphene sheet, which is 0.3812 Å^−2^ [7].

The suction energy is first studied to determine the critical radius at the neck of catenoid for the encapsulation to occur. At zero suction energy, the molecule can enter the neck of catenoid and it can move along the central axis of catenoid because the energy level inside the catenoid is lower than that of outside the catenoid. We define the neck radius at zero suction energy as *a*_0_, and the neck radius at the maximum suction energy is defined by *a*_*max*_.

### 4.1 Interaction energy between atom and catenoid

**Fig 5:**
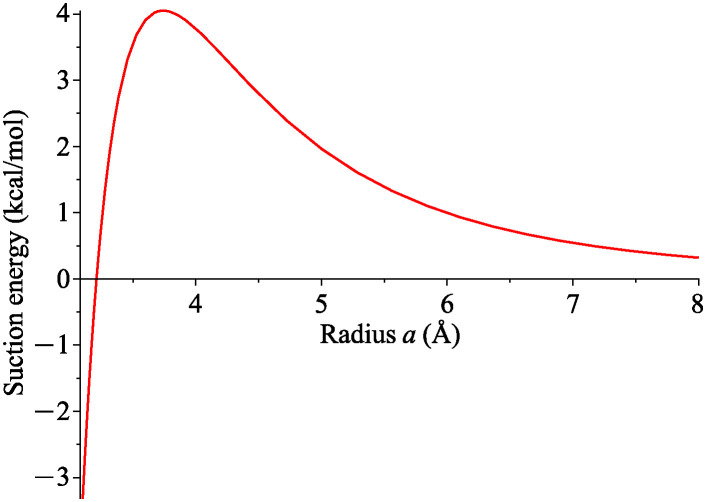
Suction energy between carbon atom and various catenoid of neck’s radii *a* and length *L* = 10 Å. This suction energy represents total work done by axial force upon atom entering catenoid.

The suction energy of a carbon atom and a catenoid as a function of the neck radius *a* is shown in Fig 5, noting that the atom is energetically favorable when the suction energy is positive. The suction energy is zero when *a*_0_ = 3.2097 Å and the maximum energy occurs when *a*_*max*_ = 3.7395 Å, and they are independent of the length *L*.

**Fig 6:**
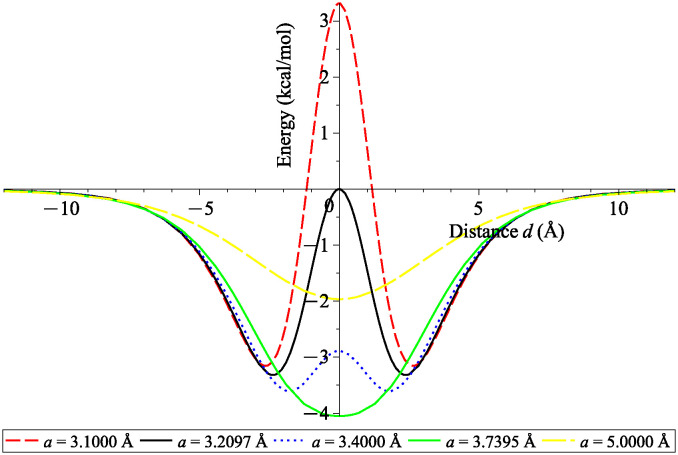
Interaction energies between carbon atom and various sizes of catenoids with neck’s radii *a* = 3.1000, 3.2097, 3.4000, 3.7395 and 5.0000 Å and length *L* = 10 Å. Note that negative energy value indicates system stability.

Fig 6 illustrates energy profiles for various sizes of *a*. We observe that when *a* < 3.7395 = *a*_*max*_, there are two local minima at equal distance from the origin due to the symmetry of the catenoid model. This implies that carbon atom prefers to move away from the centre to keep an equilibrium spacing with the surface of catenoid. However when *a* > 3.7395 = *a*_*max*_, the minimum energy occurs at *d* = 0 which means that the atom is energetically favourable at the origin. In other words, there is enough space for the atom to be at the catenoid’s neck. Noting that we observe the lowest energy level inside the catenoid when *a* = *a*_*max*_.

### 4.2 Interaction energy between fullerene and catenoid

**Fig 7:**
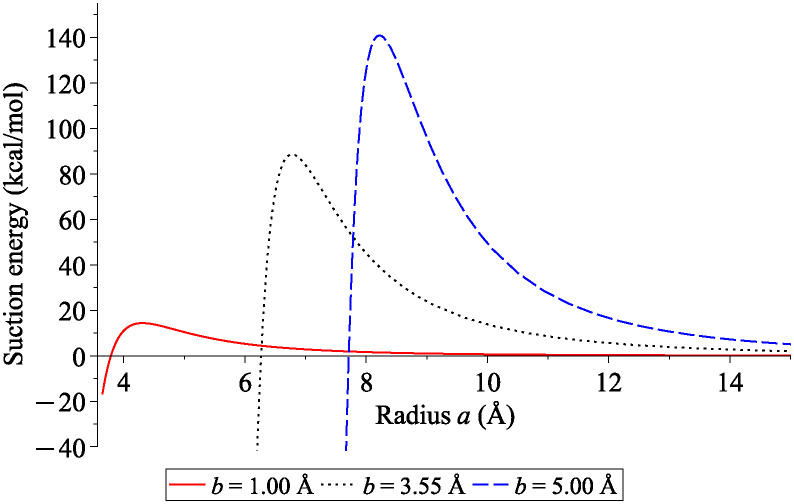
Suction energies between spheres of radii *b* = 1.00, 3.55 and 5.00 Å and catenoid with various radii *a* at the neck and length *L* = 200 Å.

Again, we begin by considering the suction energy behaviour as illustrated in Fig 7. The neck radius of catenoid at zero suction energy (*a*_0_) and that at the maximum suction energy (*a*_*max*_) are numerically determined and reported in Table 1. We also calculate the spacing between the surface of fullerene and the surface of the catenoid at the origin as given in Table 1, where we define *ε*_0_ = *a*_0_−*b* and *ε*_*max*_ = *a*_*max*_−*b*. We find that both *ε*_0_ and *ε*_*max*_ are approximately constant for all cases considered here, and they slightly decrease when the radius of fullerene increases. This finding is independent of the length *L* of catenoid.

**Table 1:** Numerical results for the relation between radius at the catenoid’s neck *a* (Å) and radius of spherical fullerene *b* (Å) with *L* = 200 Å.

Radius of sphere *b*	Radius at neck		Spacing at origin	
	*a*_0_	*a*_*max*_	*ε*_0_	*ε*_*max*_
1.00	3.7892	4.3087	2.7892	3.3087
2.00	4.7346	5.2501	2.7346	3.2501
3.00	5.7206	6.2332	2.7206	3.2332
3.55	6.2670	6.7780	2.7170	3.2280
4.00	6.7150	7.2247	2.7150	3.2247
5.00	7.7120	8.2195	2.7120	3.2195
10.00	12.7062	13.2068	2.7064	3.2068
15.00	17.7045	18.2016	2.7045	3.2016

**Fig 8:**
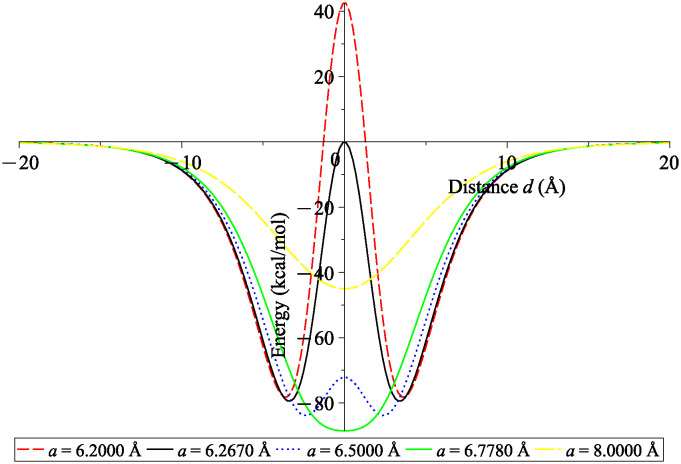
Interaction energies between fullerene of radius *b* = 3.55 Å and various sizes of catenoids with neck’s radii *a* = 6.2000, 6.2670, 6.5000, 6.7780 and 8.0000 Å, and length *L* = 200 Å. Note that negative energy value indicates system stability.

The energy profiles for the interaction between a fullerene and a catenoid are depicted in Fig 8 where the radius of the sphere is taken to be *b* = 3.55 Å to represent the case of C_60_ fullerene. Similar to the case of the atom, two minima are obtained when *a* < *a*_*max*_ whereas there is only one minimum energy location at the origin when *a* ≥ *a*_*max*_.

### 4.3 Comparison of theoretical model with numerical simulations

To compare results of the continuum model presented in this paper with numerical simulations, in LAMMPS the carbon atom is forced to move along a prescribed trajectory on the central axis of the molecular carbon catenoid, as opposed to allowing the program to determine the movement from a set of initial conditions. In other words, instead of performing molecular dynamics conventionally, we use LAMMPS for the purpose of obtaining numerical solutions. That is to calculate the energy between atom and catenoid at all points along the central axis of the catenoid in order to compare with the theoretical results for which the motion of atom is prescribed along the *z*-axis.

**Fig 9:**
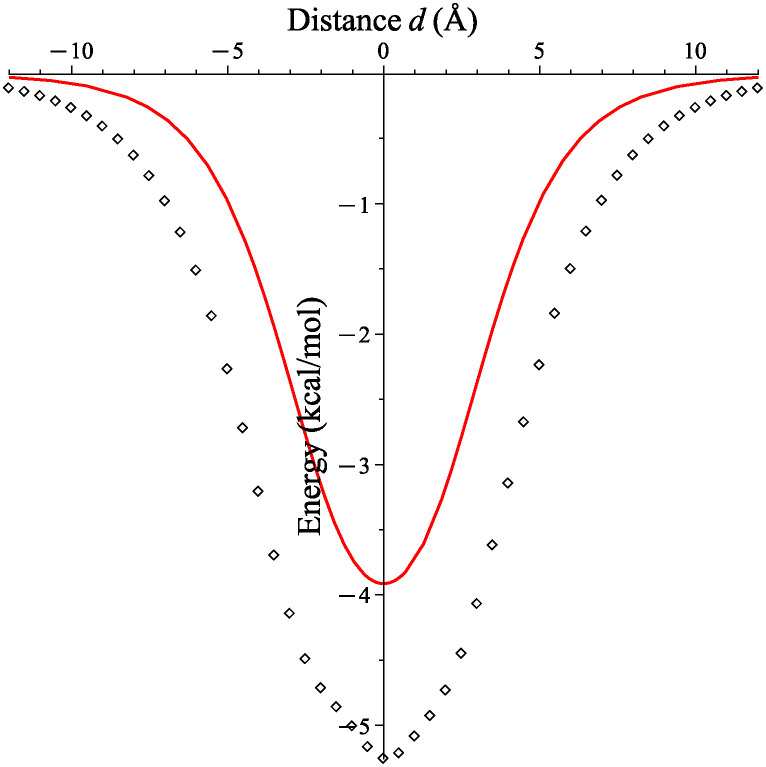
Interaction energy of a carbon atom and a catenoid with *a* = 3.9144 Å; theoretical catenoid (solid line) and molecular catenoid (dotted line).

**Fig 10:**
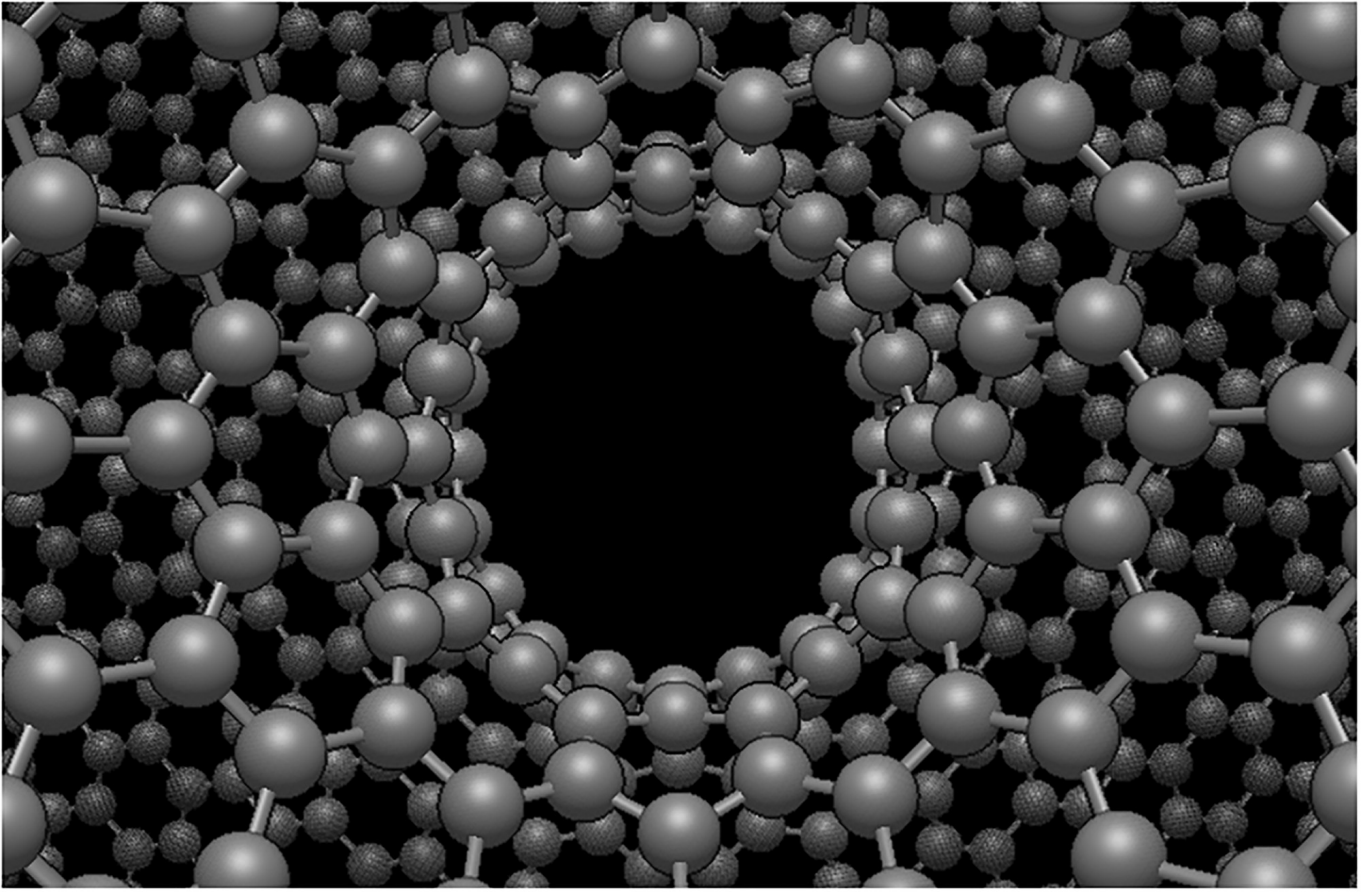
Molecular catenoid generated from Avogadro showing non-circular cross-section at its neck due to self interaction of atoms within catenoid.

The potential energy profile output from the simulated catenoid-atom interaction has the same shape as the profile of the continuum model for the theoretical catenoid of the same radius as shown in Fig 9. However, there is a consistent overestimation of the potential energy at all positions of the atom, which we suspect is due to the constructed molecular catenoid being slightly narrower than the theoretical catenoid, thus increasing the interaction strength among the structures. Furthermore, at the neck there is a slight flattening of the molecular catenoid which is due to the self attraction of atoms in the catenoid as shown in Fig 10. This flattening leads to an elliptical cross section at the neck with major and minor diameters of roughly 7.959 Å and 7.181 Å, respectively, which would further increase interaction strength due to the closer proximity of the surface with the atom. We note that the presence of a small irregularity in the profile of the simulation results, where there is a slight indentation near the origin, is simply due to the molecular catenoid not being perfectly symmetric about the *xy*−plane.

We comment that there is no direct comparison of our catenoid results with other studies in the literature. Nevertheless, the closest structures to catenoids that we can draw some comparison from are truncated carbon nanocones and hourglass-shaped channels [21, 22, 31–35]. In [32], the interaction between the atoms/fullerenes and the truncated carbon nanocone are modelled using the Lennard-Jones potential and a continuum approach. As shown in their Figs 3 and 5, the atom (fullerene) is stable within the truncated carbon nanocone with the base radius of 3.5526 Å (7.1052 Å), which aligns well with the value *a*_0_ = 3.2097 Å (6.2670 Å) obtained in our study. We comment that the study of Lee et al. [32] has motivated further investigations of truncated carbon nanocones for gas storage storage (e.g. hydrogen, methane, and neon) [33]. Additionally, Sarapat et al. [21] investigate the energetic behavior of truncated carbon nanocones interacting with water molecules, sodium, and chloride ions. Assuming these particles as points, they find the highest suction energy occurs for cone radii between 3.368 and 3.528 Å. This range is comparable to our calculated maximum radius, *a*_*max*_ = 3.7395 Å, for the case of a carbon atom interacting with a catenoid. Using a similar approach as Lee et al. [32], Ansari and Sadeghi [34] further observe asymmetric fullerene movement through truncated carbon nanocones. They find that as the cone radius decreases, the offset fullerene moves closer to the cone axis with increasing energy. This suggests that the system prefers to maintain a constant inter-molecular spacing between the fullerene and nanocone, similar to the behaviour observed here for fullerene encapsulated in catenoid.

While truncated nanocones can be compared to half of a catenoid, an hourglass-shaped channel can be considered a similar structure to a whole catenoid. That is, we have the central constriction of the hourglass, which is equivalent to the neck of a catenoid. Baowan et al. [22] study hourglass-shaped channels coated with various materials, employing both continuum approach and discrete model using LAMMPS to calculate suction energies of ions. Their findings from these two approaches are consistent. For carbon-based polymer (PAA) interacting with point charge ions, they report *a*_0_ values between 2.644 and 3.148 Å and *a*_*max*_ values between 3.080 and 3.668 Å, depending on the type of ions and the slope of the hourglass channels. Our calculated values for *a*_0_ and *a*_*max*_ are slightly larger than those reported in [22], which is likely due to the different material used, but exhibit a similar trend.

Based on these previous findings, the size of the cavity is a key factor in the encapsulation of carbon nanostructures. This fact is also supported by our results here, which indicate that the neck of catenoid plays a crucial role in allowing carbon atoms or fullerenes to be encapsulated within the structure.

## 5 Conclusion

In this paper, we construct a molecular structure of carbon catenoid in comparison with theoretical catenoid obtained from minimising the Willmore energy functional [8]. Since applications of catenoids involve molecular transport and storage, we investigate the encapsulation of atoms and spherical molecules within catenoids. We employ a continuum approach together with the Lennard-Jones potential to determine the interaction energy. The catenoid is modelled as a symmetric structure with length *L* from the origin along the positive and negative *z*-axis, and the neck of radius *a* is at the origin. Two interactions considered here are atom-catenoid and fullerene-catenoid.

In the first scenario, we consider an atom positioned along the central axis of catenoids. Integration techniques allow us to evaluate the total interaction energy analytically. Our findings indicate that the atom is energetically favourable at the origin when the catenoid’s neck radius *a* exceeds 3.7395 Å. Conversely, for neck radii smaller than 3.7395 Å, two minima at equal distance from the origin appear at the upper and lower parts of a catenoid.

Similar energy behaviour is observed for the interaction between a fullerene of radius *b* and the catenoid. Interestingly, the closest distance between the fullerene’s surface and the catenoid’s surface denoted by *ε* = *a* − *b* remains approximately constant for all sizes of fullerenes considered. For encapsulation to occur, the system requires *ε* to be greater than 2.7892 Å. To achieve maximum suction energy, *ε* needs to be larger than 3.3087 Å.

The validity of our mathematical model is confirmed through numerical simulations. We construct a discrete catenoid structure and use LAMMPS to evaluate the interaction energy between the catenoid and a carbon atom. The potential energy profile obtained from the simulated catenoid-atom interaction is in reasonable agreement with the profile of the continuum model for a catenoid of the same radius. The study shown in this paper demonstrates a proof of concept to construct a molecular carbon catenoid and provides a basis for the design of irregularly shaped channels using catenoids for gas storage, drug delivery systems and ion transport channels.

Finally, we summarise the main findings of our study as follows:

- A molecular carbon catenoid is proposed and we use Avogadro software to construct the structure in order to compare with a theoretical catenoid which is an absolute minimiser of the Willmore energy functional.
- A continuum approach using the Lennard-Jones potential is adopted to determine the energy between a theoretical catenoid interacting with an atom and a fullerene.
- An atom is most stable at the centre of the catenoid when the neck radius is 3.7395 Å.
- Encapsulation of fullerenes of various radii requires the inter-molecular spacing between the surfaces of fullerene and catenoid to be greater than 2.7892 Å, and the maximum suction energy is attained when this distance surpasses 3.3087 Å, irrespective of the fullerene radius.
- Numerical simulations performed in LAMMPS for a molecular catenoid are in good agreement with the continuum model for a theoretical catenoid.
- Encapsulation of atoms and fullerenes within catenoids is purposely investigated here to explore potential uses of catenoids in designing molecular structures for gas storage, drug delivery, and ion transport channels.

## Supporting information

S1 File Construction of a molecular catenoid using Avogadro. (PDF)
