# Supplementary material for: Exploring carbon catenoids and their applications for encapsulation of carbon nanostructures
Source: PLoS One. 2024 Sep 26;19(9):e0310740. doi: 10.1371/journal.pone.0310740 (PMC11426522; doi:10.1371/journal.pone.0310740)
Supplement: S1 File — (PDF) [file pone.0310740.s001.pdf]

Supporting Information - Exploring Carbon Catenoids and Their Applications for Encapsulation of Carbon Nanostructures

September 9, 2024

1 Construction of a molecular catenoid using Avogadro

Due to the symmetry of a catenoid, we only need to construct half of a catenoid in Avogadro, then replicate the structure to generate another half of the catenoid. Both parts are then joined where the geometry of the structure is optimised by Avogadro using an inbuilt extension called Open Babel.

Next, we describe steps to construct half of a catenoid from a section of $(n, 0)$ carbon nanotube. Figure 1 illustrates each step in the construction graphically on a starter from a (10,0) nanotube.

1. Starting with a ring section of $(n, 0)$ nanotube comprising all hexagonal network,

2. Adding the next ring section comprising 4 heptagons evenly spaced among $n - 4$ hexagons,

3. Adding the next ring section containing all hexagons,

4. Adding the next ring section containing 4 heptagons spaced evenly among hexagons and in the middle of the heptagons introduced in Step 2,

5. Adding the next ring section containing hexagons and 4 pentagons. The 4 pentagons are to be connected directly to the heptagons introduced in Step 4,

6. Adding the next ring section containing all hexagons,

7. Repeating Steps 2-6 if required.

When the number of hexagons in the starter ring is not evenly divided by four, the implementation of Steps 2 to 6 become staggered, with heptagons being introduced only when they would be directly between two of the initial four heptagons. This staggering is seen in Fig. 1, where only two heptagons are introduced in Fig. 1.4, and the next two are added in Fig. 1.5. We note that after each step when the next ring section is added the geometry of the structure is optimised by Avogadro using an inbuilt extension Open Babel.

We can also follow the above processes to generate larger catenoids by starting with $(13, 0)$ and $(20, 0)$ carbon nanotube sections as shown in Fig. 2. In Figs. 1 and 2, the heptagonal and pentagonal lattices are highlighted in blue.

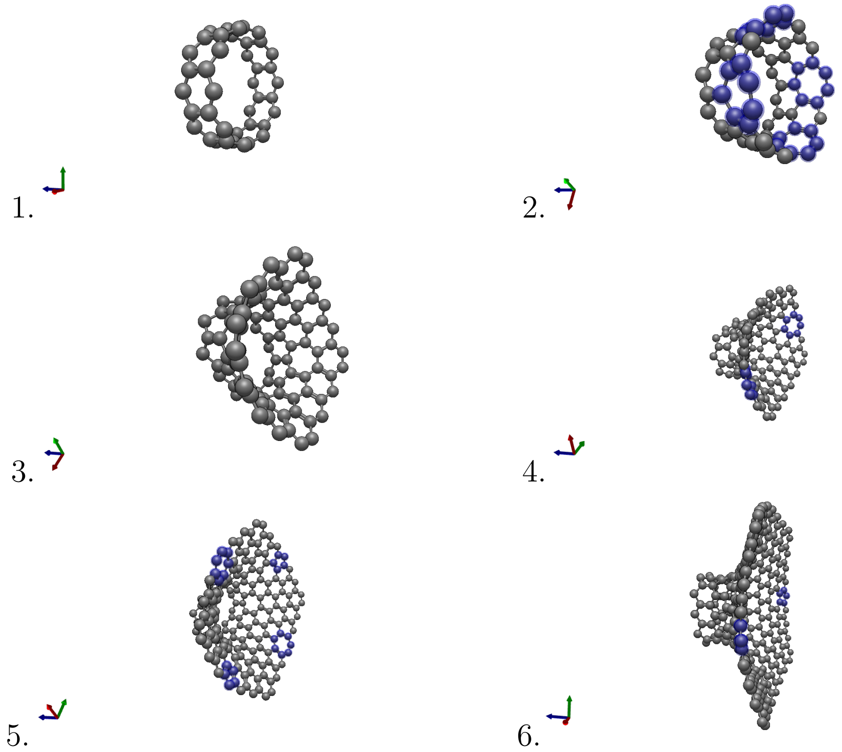

Figure 1: Steps involved in constructing a molecular carbon catenoid. Highlighting in blue indicates the locations of heptagons and pentagons on the catenoids.

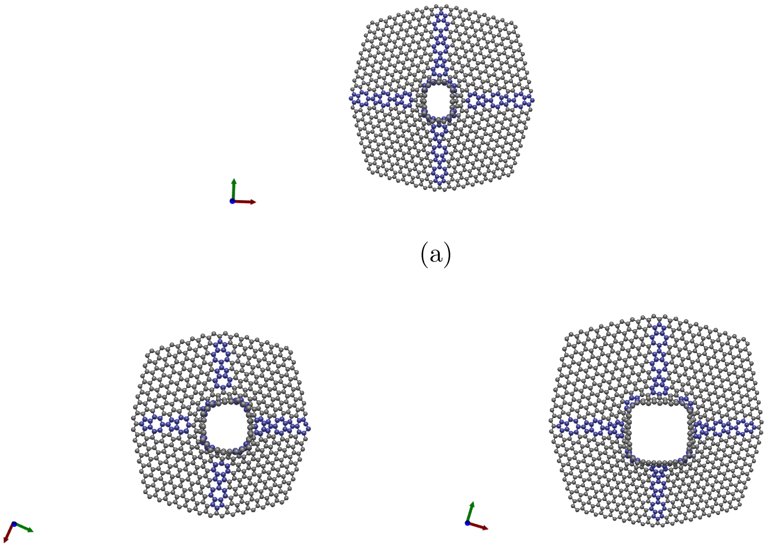

(a) (b) (c)

Figure 2: Half of a catenoid structure constructing from a section of carbon nanotube $(n, 0)$: (a) $(10, 0)$, (b) $(13, 0)$ and (c) $(20, 0)$. Highlighting in blue indicates the locations of heptagons and pentagons on the catenoids.
